# Adaptation of the Phytopathogenic Fungus *Microdochium nivale* to the Fungicides Tebuconazole and Fludioxonil

**DOI:** 10.3390/jof11120859

**Published:** 2025-12-02

**Authors:** Olga A. Gogoleva, Guzalia Sh. Murzagulova, Egor A. Ryazanov, Maria I. Antonova, Anastasiya A. Lebedeva, Sergey N. Ponomarev, Mira L. Ponomareva, Vladimir Y. Gorshkov

**Affiliations:** 1 Federal Research Center “Kazan Scientific Center of the Russian Academy of Sciences”, 420111 Kazan, Russiaantonovami.work@gmail.com (M.I.A.);; 2 Institute of Fundamental Medicine and Biology, Kazan Federal University, 420008 Kazan, Russia

**Keywords:** fungicide resistance, intrapopulation heterogeneity of phytopathogenic fungi, *Microdochium nivale*, snow mold, virulence, stress-induced genome modification, plant infectious diseases

## Abstract

The study investigated the adaptation of the snow mold causal fungus, *Microdochium nivale*, to the fungicides fludioxonil and tebuconazole. Analysis of intrapopulation diversity among 136 *M. nivale* strains from two Russian populations revealed no strains with high-level resistance to these fungicides. However, the strains exhibited considerable variability in their sensitivity to small fungicide doses. Fungicide sensitivity levels were not associated with virulence levels, whereas strains from different phylogenetic groups exhibited different predispositions to decreased sensitivity to tebuconazole and fludioxonil. In vitro adaptation experiments were conducted to assess: (1) the potential ability of *M. nivale* to acquire high-level resistance to these fungicides; (2) the relative adaptation efficiency to each fungicide; and (3) the impact of resistance acquisition on virulence. Our results showed that *M. nivale* strains could adapt to high concentrations of both fungicides with little or no effect on virulence. Adaptation to fludioxonil was significantly less effective than to tebuconazole. To get closer to understanding the mechanisms of fludioxonil adaptation in *M. nivale*, whole-genome sequencing was performed on a fludioxonil-adapted derivative and its parental fludioxonil-sensitive strain. Comparative genome analysis identified mutations potentially involved in the enhanced fludioxonil resistance, which are discussed within the framework of molecular resistance mechanisms.

## 1. Introduction

Plant diseases caused by phytopathogenic fungi lead to significant yield losses worldwide. To reduce such losses, fungicides are widely applied. However, phytopathogenic fungi rapidly adapt to fungicides, posing an additional food security threat [1]. Therefore, rational fungicide management represents an important challenge in modern agriculture, and effectively addressing it requires conducting in-depth studies on fungicide resistance among phytopathogenic fungi.

Among the most known mechanisms of fungicide resistance are: (1) point mutations in the gene encoding the fungicide’s target protein, which reduce the binding affinity of the fungicide; (2) duplication or upregulation of the target gene, leading to elevated amounts of the target protein; (3) enhanced synthesis of transporter proteins that actively export the fungicide out of the fungal cell; (4) enzymatic modification or detoxification of the fungicide inside the fungal cell [2,3,4]. These mechanisms may confer varying levels of resistance, ranging from qualitative resistance, where even high doses of fungicide (commonly used in agricultural practice) cause no or minimal effect on fungal growth, to quantitative resistance, where the sensitivity threshold increases, making the fungus less sensitive to the fungicide [5]. Given that fungicide concentration is uneven in the treated agrocenosis, a decrease in sensitivity to fungicide may provide an advantage for less sensitive fungal strains over more sensitive ones, even though full fungicide resistance is not yet achieved [3].

Many fungicides used in agriculture belong to the azole and phenylpyrrole classes. Azoles inhibit sterol demethylation by binding to their target enzyme in fungal cells—lanosterol-14α-demethylase (CYP51)—thereby disrupting ergosterol synthesis, impairing membrane function, and ultimately leading to cell death [6]. Adaptation to different azole fungicides is primarily related to mutations in CYP51; whereby, mutations in different sites of this gene are necessary to confer resistance to different azole fungicides [7,8]. In addition, the elevation of resistance levels to azole fungicides is associated with increased expression of the CYP51 gene and/or genes encoding multidrug resistance transporters [9,10]. The combination of different adaptation-related features (mutations and gene expression regulation) determines the resulting level of resistance or sensitivity to particular azoles [7,8,11]. In the last decade, an increase in resistance levels to azole fungicides has been observed in populations of such phytopathogens as *Zymoseptoria tritici* [8,10,11], *Mycosphaerella graminicola* [6], *Fusarium graminearum* [12], and *Botrytis cinerea* [13].

The mechanism of action of phenylpyrrole fungicides, including their most commonly used representative, fludioxonil, remains relatively less understood. Fludioxonil targets group III hybrid histidine kinases (HHKs), overstimulating the high-osmolarity glycerol (HOG) pathway to induce hyphal swelling and bursting [14,15]. Two major mechanisms of fludioxonil resistance are highlighted: (1) mutations in group III HHKs or downstream kinases (Os-1, Os-4, Os-5, Os-2); and (2) overproduction of transporters that efflux the fungicide [16,17,18,19,20,21,22]. The mechanism of fludioxonil resistance has been best described in a recent study on *Fusarium graminearum*, where mutation-driven modifications of the histidine kinase FgOs1 reduce its affinity for fludioxonil, conferring resistance to this fungicide. However, the cost of this fungicide resistance appeared to compromise fungal fitness [23]. The association between fludioxonil resistance formation and compromised fungal fitness aligns with the fact that this fungicide is regarded as a “low-risk fungicide”, meaning that adaptation to it is a rare phenomenon [15,24]. Nevertheless, increased resistance to fludioxonil is still found in populations of phytopathogenic fungi such as *Botrytis cinerea* and *Fusarium graminearum*, with different mutations associated with elevated fludioxonil resistance: overproduction of major facilitator superfamily (MFS) transporters in *B. cinerea* [25] and mutations in HHKs in *F. graminearum* [23].

Mutations conferring fungicide resistance may have pleiotropic effects on other phenotypic traits in fungi, including virulence [8,20]. Adaptation to the phenylpyrrole fungicide fludioxonil or azole fungicide tebuconazole has been more often reported to be associated with a reduction in virulence [20,25,26,27]. However, cases where fludioxonil- or tebuconazole-resistant strains were as virulent as fungicide-sensitive ones have also been described [28,29,30]. Herewith, even within a particular fungal species, different studies show the differential effect of the acquisition of fludioxonil or tebuconazole resistance on virulence [26,28,31,32,33,34,35].

The issue of fungicide resistance is especially critical concerning fungi that parasitize winter crops beneath the snow cover or shortly after it melts [36]. Diseases caused by such psychrophilic or psychrotolerant pathogens are known as snow molds. Since the snow cover prevents fungicide application, fungicides to reduce snow mold damage can only be used preventively, before the snow cover forms. Therefore, the fungicide concentration during disease progression may decrease over time due to partial degradation and leaching. In turn, reduced fungicide doses facilitate more effective fungal adaptation and increase the survival probability of strains with partial resistance (i.e., less sensitive strains), even if full resistance is not achieved.

The most widespread snow mold pathogen in Northern Europe, including the European part of Russia where winter crops are extensively cultivated, is *Microdochium nivale* [36,37]. Populations of *M. nivale* have recently been shown to contain a high proportion of strains with full resistance to the benzimidazole fungicide carbendazim [38]. Additionally, different strains of *M. nivale* have been shown to exhibit variable sensitivity to other fungicides [39,40,41,42,43,44,45,46,47]. However, the intrapopulation variability of strains in the sensitivity to fungicides (except carbendazim) has not been investigated. Therefore, our study aimed to analyze the intrapopulation variability of *M. nivale* strains regarding their resistance or sensitivity to the azole fungicide tebuconazole and the phenylpyrrole fungicide fludioxonil—both recommended for suppressing snow mold—and to assess possible relationships between resistance or sensitivity to these fungicides and other strain traits. We also attempted to deepen insights into the mechanisms of phenylpyrrole resistance, which remain poorly understood to date.

## 2. Materials and Methods

### 2.1. Strains Used in This Study

The 136 *M. nivale* strains used in this study were isolated from winter cereal crops: winter rye (*Secale cereale* L. cv. Ogonek), winter wheat (*Triticum aestivum* L. cv. Nadezhda), and winter triticale (×*Triticosecale* Wittm. cv. Kornet), as described in our previous study [48]. The plants from which the *M. nivale* strains were isolated were grown in competitive variety field trials in two agrocenoses located 59 km apart in a straight line within the Republic of Tatarstan: (1) in the Laishevo district (latitude 55.625164 N, longitude 49.351334 E) and (2) in the Arsk district (latitude 56.113468 N, longitude 49.774500 E).

### 2.2. Evaluation of Fungicide Resistance/Sensitivity of the Strains

To screen *M. nivale* strains for resistance/sensitivity to fludioxonil and tebuconazole, the fludioxonil-formulated fungicide Maksim (AgroExpertGroup, Moscow, Russia) tebuconazole-formulated fungicide Tebu-60 (AgroExpertGroup, Moscow, Russia) were used. Either fludioxonil or tebuconazole were added to potato-sucrose agar (potato decoction (200 g of potato tubers per one liter of water), sucrose 30 g L^−1^, 2% agar, pH 6.2) supplemented with 200 µg/mL gentamicin (PSA). *M. nivale* strains were grown on PSA both without the fungicides (control) and with fungicides at concentrations of 1.0 × 10^−3^ M (which corresponds to 248.2 µg mL^−1^ of fludioxonil or 307.8 µg mL^−1^ of tebuconazole), 1.0 × 10^−4^ M, 5.0 × 10^−5^, 2.5 × 10^−5^, 1.0 × 10^−5^, 5.0 × 10^−6^. To assess the effect of the fungicides on the studied strains, 7-mm-diameter mycelial plugs, cut from the periphery of 10-day-old cultures of the strains, were placed at the center of Petri dishes containing PSA and PSA supplemented with either fludioxonil or tebuconazole. The fungi were cultured in darkness at 20 °C for 2 weeks. Each day, two perpendicular radii of each fungal colony were measured, and their average was used to estimate the fungal growth rate. The growth rate was extrapolated from the slope of the linear part of the growth curve. Each strain was analyzed in three biological replicates under each condition (without and with each fungicide). The effect of each fungicide was expressed as the percentage of growth inhibition observed in its presence. The effective concentrations (EC_90_) of tebuconazole and fludioxonil that cause 90% growth inhibition were calculated using OriginPro 8.0 (OriginLab Corporation, Northampton, MA, USA).

### 2.3. Assessment of Virulence and Genetic Traits of the Strains

The assessment of virulence of the 136 studied *M. nivale* strains, as well as the sequencing of the internal transcribed spacer 2 (ITS2), elongation factor 1α (EF-1α), and β-tubulin (β-Tub) genes, was performed in our previous study [48]. Here, these data were used to analyze whether relationships existed between resistance or sensitivity to fludioxonil and tebuconazole (analyzed in this study) and virulence or genetic traits (analyzed in a previous study) in the studied strains. The virulence of the strains was previously analyzed on aseptically grown winter rye cv. Ogonek, winter wheat cv. Nadezhda, and winter triticale cv. Kornet in vitro [48]. Seeds sterilized with a 5% AgNO_3_ solution were germinated for 36 h at 28 °C on ¼ diluted Murashige and Skoog medium without organic carbon (¼ MS). Seedlings were transferred to individual sterile 50 mL glass tubes containing 10 mL of ¼ MS and were infected 12 h later. For plant inoculation, an 8 mm mycelial plug cut from the periphery of 7- to 10-day-old cultures grown on PSA (or a sterile PSA plug for mock-inoculation) was placed in contact with the seedling. Plants were grown at 20 °C with a 16 h light/8 h dark cycle for 20 days. The virulence of the strains was analyzed in at least 20 biological replicates and expressed as reduced root dry weight (RRDW, %) of infected plants compared to control plants, since this parameter allows for more precise differentiation of strains based on their effect on the host plant than visual symptom assessment [37]; a greater reduction in root dry weight indicates higher strain virulence.

The nucleotide sequences of the ITS2, the EF-1α fragment, and the β-Tub fragment from the 136 studied *M. nivale* strains were determined by amplicon sequencing in our previous study [48]. For each of the 136 strains, the target sequences were amplified using primers ITS3_KYO2: 5′–GAT GAA GAA CGY AGY RAA–3′ and ITS4: 5′–TCC TCC GCT TAT TGA TAT GC–3′ [49]; MicEF_F1: 5′–GGT GAG TTC GAG GCT GGT ATC–3′ and MicEF_R1: 5′–CAG GGG GCG TTG GTG G–3′ [48]; MicTub_F1: 5′–ACG CTC CTC ATC TCC AAG ATC–3′, MicTub_R1: 5′–GAA ACG CAG GCA GGT GGT–3′ [48], respectively. DNA libraries were prepared according to the Illumina protocol (Illumina protocol, part no. 15044223, Rev. B). The indexing of libraries was performed using the Nextera XT Index Kit v2 (Illumina, San Diego, CA, USA). The libraries were sequenced using the MiSeq Reagent Kit v3 (600-cycles) (Illumina). The sequences were deposited in the NCBI database under the following accession numbers: PQ516916.1 and PQ516917.1 (“A” and “B” ITS2 sequence variants, respectively, revealed among the studied strains), PQ538502.1 and PQ538503.1 (“A” and “B” EF-1α sequence variants, respectively, revealed among the studied strains), and PQ538504, PQ538505, and PQ538506 (“A”, “B”, and “C” β-Tub sequence variants, respectively, revealed among the studied strains).

### 2.4. In Vitro Adaptation of M. nivale Strains to Fludioxonil and Tebuconazole

For in vitro adaptation of *M. nivale* to fludioxonil and tebuconazole, the most sensitive strains that displayed complete growth inhibition at a concentration of 1.0 × 10^−6^ M were selected. Strains were cultured in liquid potato-sucrose medium with aeration at 120 rpm and 20 °C in the presence of 1.0 × 10^−7^ M fludioxonil or tebuconazole. During each passage, the mycelial pellet was transferred to fresh medium containing either the initial fungicide concentration or higher concentrations (1.0 × 10^−6^, 1.0 × 10^−5^, 2.0 × 10^−5^, 3.0 × 10^−5^, 4.0 × 10^−5^, 5.0 × 10^−5^, 6.0 × 10^−5^, 7.0 × 10^−5^ M). As the strains gradually adapted, fungicide concentrations were incrementally increased, and the relationships between the fungicide concentrations at which each strain could grow and the number of passages required to acquire this ability were plotted, reflecting the adaptation curves of the strains. Following 20 passages, the areas under the adaptation curves, which reflect the adaptation coefficients, were calculated for each strain using the formula:

$$AC=\sum_{i=1}^{20}\left(\frac{C_{i}+C_{i-1}}{2}\right)*1000$$

where *AC* is the adaptation coefficient, *i* is the passage number, and *C_i_* is the maximal fungicide concentration at which a strain could grow at passage *i.*

### 2.5. Evaluation of Virulence of Fungicide-Adapted Strains

The virulence of the obtained in vitro fungicide-adapted derivative *M. nivale* strains was compared with that of their parental fungicide-sensitive strains stored as museum cultures, as well as with that of the parental fungicide-sensitive strains that, similar to the fungicide-adapted derivatives, were consistently cultured for 20 passages in vitro but without fungicides (passaged strains). The virulence of the strains was analyzed on aseptically grown winter rye cv. Ogonek, as described above (Section 2.3).

### 2.6. Statistics

To analyze the significance of differences in quantitative traits between different samples, the Mann-Whitney U test was used (with *p* < 0.05 indicating significance); for comparisons involving more than two samples, the Bonferroni correction for multiple comparisons was applied (with FDR < 0.05 indicating significance). Relationships between quantitative traits were assessed using Spearman’s rank correlation, with *p* < 0.05 considered indicative of a significant correlation.

### 2.7. Genome Assembly, Analysis and Annotation

The mycelium of the *M. nivale* strain F_00246, grown in liquid potato-sucrose medium, was sent to the commercial company LLC Algaevitapro (Moscow, Russia) for DNA extraction (SMRTbell prep kit 3.0 (PacBio)) and sequencing using the PacBio HiFi platform (Pacific Biosciences, Menlo Park, CA, USA). The obtained PacBio HiFi reads (available at the NCBI BioProject PRJNA1356363) were initially characterized using SeqKit [50] and then filtered with Filtlong [51] to enrich for high-quality data, retaining sequences with a minimum length of 2000 bp and preferentially keeping the highest-quality reads. Genome size was estimated from k-mer spectra using Merqury v 1.3 [52] and GenomeScope2 [53] across k-mer sizes k = 21 to k = 31; the resulting estimate was used as input for the genome assembler. De novo genome assembly was performed with Hifiasm [54] using parameters −k19 (−k17), −w19 (−w17), and −D4 together with the computed genome size. Multiple assemblies generated under varying parameter settings were benchmarked with QUAST v. 5.3.0 [55] to guide selection of the optimal genome assembly. The genome assembly consensus quality (QV) was quantified using Merqury [52]. Genome completeness was assessed using BUSCO v 6.0.0 [56] with various gene predictors, including “euk_genome_aug”, “euk_genome_met”, and “euk_genome_min”. Structural and functional annotation of the *M. nivale* strain F_00246 genome assembly was performed using the FUNANNOTATE pipeline [57], configured and trained with incorporated data from the structural and functional annotation of the previously analyzed *M. nivale* strain F_00608 genome [58] to improve gene model prediction and support functional assignment. The genome assembly has been deposited at GenBank as part of the NCBI BioProject PRJNA1356363. The assembly can alternatively be accessed at [59].

### 2.8. Searching for Fludioxonil-Induced Mutations

The mycelium of the *M. nivale* strain F_00246-FR (an in vitro-adapted derivative of the F_00246 strain), grown in liquid potato-sucrose medium supplemented with 5 × 10^−4^ M fludioxonil, was sent to the commercial company LLC Algaevitapro (Moscow, Russia) for DNA extraction (Illumina DNA Prep, (M) Tagmentation) and sequencing using the Illumina NovaSeq X Plus platform. Illumina reads were quality checked with FastQC [60] to evaluate per-base sequence quality, GC content, adapter content, and other standard metrics. Reads were filtered and trimmed with fastp [61] to remove adapters, low-quality bases, and short fragments, yielding clean FASTQ files. Filtered reads were aligned to the assembled genome of the *M. nivale* strain F_00246 (Section 2.7) using BWA-MEM2 [62]. Alignments were processed with SAMtools [63] to sort the BAM file and perform deduplication.

Identification of mutations (single or multiple nucleotide polymorphisms (SNPs or MNPs), insertions, and deletions) in the fludioxonil-adapted *M. nivale* strain F_00246-FR compared to its parental natural fludioxonil-sensitive strain F_00246 (Section 2.7), as well as filtering of mutations, were carried out using two tools: (1) GATK 4 [64] with the following thresholds: for SNPs—QD < 2.0, QUAL < 30.0, SOR > 3.0, FS > 60.0, MQ < 40.0, MQRankSum < −12.5, ReadPosRankSum < −8.0; for indels—QD < 2.0, QUAL < 30.0, FS > 200.0, ReadPosRankSum < −20.0; and (2) Strelka2 [65] with default filter annotations. Resulting VCF files (outputs from GATK 4 and Strelka2) were normalized and left-aligned relative to the genome assembly of the parental natural fludioxonil-sensitive strain F_00246 using BCFtools [66]. The mutations identified using GATK 4 and Strelka2 were compared using custom Python v 3.13.17 scripts, and mutations present in both call sets were considered as the consensus set representing mutations associated with *M. nivale* adaptation to fludioxonil. The identified mutations were mapped to the *M. nivale* F_00246 genome to determine their locations (CDS, introns, intergenic spacers) using custom Python scripts. The functions of the particular genes where the mutations were observed were derived from the annotation of the reference genome of *M. nivale* F_00608 [58].

## 3. Results

### 3.1. Differential Sensitivity of Microdochium nivale Strains to Tebuconazole and Fludioxonil

The growth of all 136 tested strains was completely inhibited by 10^−3^ M and 10^−4^ M concentrations of tebuconazole and fludioxonil, indicating that they are sensitive to the fungicide doses typically recommended for plant protection. To assess potential differences in strain sensitivity to fungicides, we first randomly selected 25 strains to analyze their growth at different fungicide concentrations: 5.0 × 10^−6^, 1.0 × 10^−5^, 2.5 × 10^−5^, 5.0 × 10^−5^ M. The effective concentrations (EC_90_) of tebuconazole and fludioxonil causing 90% growth inhibition in the 25 studied strains were estimated at 1.9 × 10^−5^ M (5.9 μg mL^−1^) and 3.7 × 10^−5^ M (9.2 μg mL^−1^), respectively. At concentrations of 1.0 × 10^−5^ M and below for tebuconazole and fludioxonil, all 25 strains displayed growth, although significantly reduced compared to growth without fungicides, whereas at 2.5 × 10^−5^ M and above, complete growth inhibition was observed in some strains. Therefore, the 2.5 × 10^−5^ M concentration was chosen to screen all 136 strains for fungicide sensitivity, as it was the lowest concentration at which some strains showed complete growth inhibition, allowing differentiation between fully sensitive and less sensitive strains.

**Figure 1:**
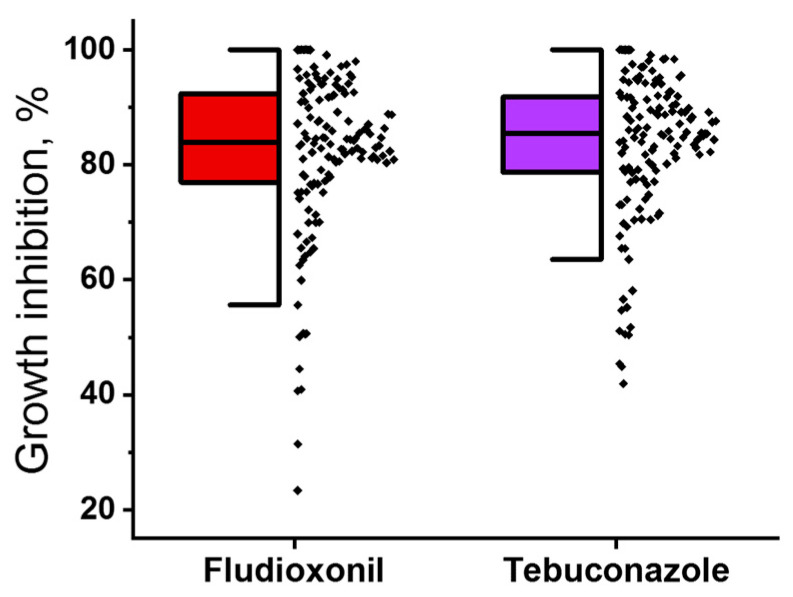
Distribution of sensitivity levels to fludioxonil (red) and tebuconazole (purple) among 136 *Microdochium nivale* strains. The sensitivity level of each strain was determined as the percentage of growth inhibition observed in the presence of 2.5 × 10^−5^ M fludioxonil or tebuconazole, compared to growth in the absence of fungicides.

Among 136 strains, the growth of eight strains was completely inhibited by 2.5 × 10^−5^ M fludioxonil, while the growth inhibition for the remaining 128 strains ranged from 23.3% to 99.1%. Tebuconazole at 2.5 × 10^−5^ M completely inhibited the growth of seven strains, whereas the growth inhibition for the other strains ranged from 42.0% to 99.1%. The sensitivity levels among the strains in the pool showed a continuous distribution (Figure 1).

### 3.2. Analysis of Potential Relationships Between Fludioxonil or Tebuconazole Resistance and Various Phenotypic and Genetic Traits of M. nivale Strains

A small but significant correlation was observed between strain sensitivity levels to fludioxonil and tebuconazole (Spearman correlation coefficient 0.28, *p* < 0.05) (File S1). Sensitivity levels to fludioxonil and tebuconazole did not differ significantly between strains from different agrocenoses (File S2). No relationships were observed between the levels of strain sensitivity to fludioxonil or tebuconazole and the virulence of the strains on each of the three winter crops (rye, wheat, and triticale), nor between fungicide sensitivity and the origin of the strains (crop or plant part from which a strain was isolated) (File S3).

**Figure 2:**
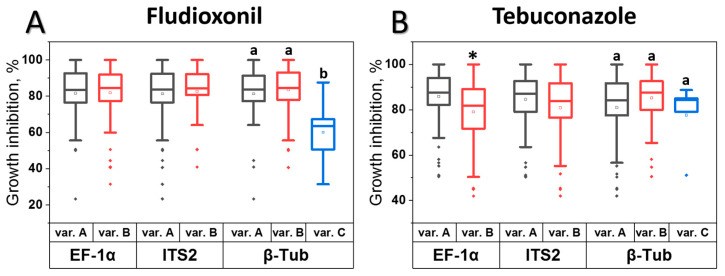
Analysis of the association between sensitivity levels to fludioxonil (**A**) or tebuconazole (**B**) and variants in the nucleotide sequences of three barcodes: the internal transcribed spacer 2 (ITS2) (A or B variant), the elongation factor 1α (EF-1α) gene (A or B variant), and the β-tubulin (β-Tub) gene (A, B, or C variant) in 136 *Microdochium nivale* strains. The sensitivity level of each strain was determined as the percentage of growth inhibition observed in the presence of 2.5 × 10^−5^ M fludioxonil or tebuconazole, compared to growth in the absence of fungicides. Asterisks or different letters above the bars indicate significant differences in sensitivity levels between strains with different barcode variants (Mann-Whitney test, *p* < 0.05, or Mann-Whitney test with Bonferroni correction for multiple comparisons, FDR < 0.05).

The *M. nivale* strains analyzed in this study were previously classified into distinct phylogenetic groups based on variations in the nucleotide sequences of three barcodes: the internal transcribed spacer 2 (ITS2), elongation factor 1α (EF-1α), and β-tubulin (β-Tub) [48]. Among the 136 studied strains, each strain had one of two types of ITS2 sequences (“A” (87 strains) or “B” (49 strains)), one of two types of EF-1α sequences (“A” (83 strains) or “B” (53 strains)), and one of three types of β-Tub sequences (“A” (54 strains), “B” (77 strains), or “C” (5 strains)); accordingly, the phylogenetic strain groups were differentiated [48]. In the present study, we investigated the potential relationships between phylogenetic group classification (barcode variants) and the sensitivity levels of strains to fludioxonil and tebuconazole. No significant relationship was observed between ITS2 variants (PQ516916.1 (“A” variant) and PQ516917.1 (“B” variant)) and sensitivity to either fungicide (Figure 2). In turn, strains carrying the “B” variant of EF-1α (PQ538503.1) were significantly less sensitive to tebuconazole than those with the “A” variant (PQ538502.1), while strains with the “C” variant of β-Tub (PQ538506) exhibited lower sensitivity to fludioxonil compared to strains carrying the “A” and “B” variants (PQ538504 and PQ538505, respectively) (Figure 2).

### 3.3. Adaptation of M. nivale Strains to Tebuconazole and Fludioxonil In Vitro

**Figure 3:**
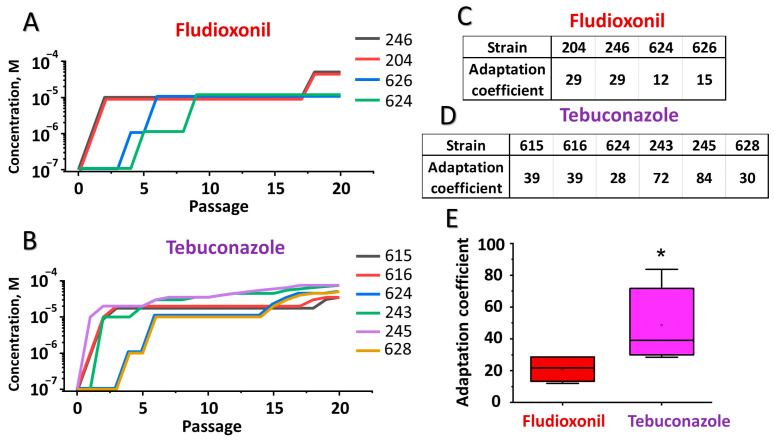
In vitro adaptation of *Microdochium nivale* strains to fludioxonil and tebuconazole. Sections (**A**,**B**) show the adaptation curves (the relationships between the fungicide concentrations at which each strain could grow and the number of passages required to acquire this ability) of the strains to fludioxonil and tebuconazole, respectively. Strains are indicated by the last three digits of the strain’s accession number. Sections (**C**,**D**) show the adaptation coefficient values (areas under the adaptation curves) for strains adapted to fludioxonil and tebuconazole, respectively. Section (**E**) shows the distribution of adaptation coefficient values for strains adapted to fludioxonil and tebuconazole; an asterisk indicates a significant difference between adaptation coefficients for the two fungicides (Mann-Whitney test, *p* < 0.05).

Since full resistance to both fludioxonil and tebuconazole has not been observed among the studied *M. nivale* strains, we investigated whether *M. nivale* is, in principle, able to acquire full resistance to these fungicides and compared the efficiency of adaptation to each. For this purpose, the most fungicide-sensitive *M. nivale* strains were consistently cultured at increasing concentrations of the fungicides. The relationships between the fungicide concentrations at which each strain could grow and the number of passages required to acquire this ability were plotted, reflecting the adaptation curves of each strain. From two to eight passages were required for the strains to acquire the ability to grow at a concentration of 10^−5^ M fungicide (Figure 3). Adaptation to higher fungicide concentrations required more passages. The acquired fungicide-resistant phenotype was maintained even after the adapted strains were cultured for 10 passages in vitro without fungicides. Following 20 passages of adaptation, the areas under the adaptation curves, reflecting the adaptation coefficient, were calculated for each strain and compared between strains adapted to different fungicides. The adaptation coefficient was significantly higher for strains adapted to tebuconazole compared to those adapted to fludioxonil (Figure 3), indicating that adaptation of *M. nivale* to tebuconazole proceeded more effectively than to fludioxonil.

**Figure 4:**
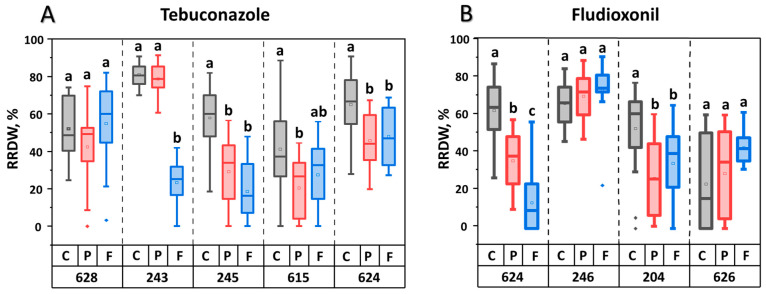
Virulence of natural *Microdochium nivale* strains (gray bars, control, C), their derivatives that were consistently cultured in vitro for 20 passages (red bars, passaged, P), and their tebuconazole- (**A**) or fludioxonil- (**B**) adapted derivatives that were consistently cultured in vitro for 20 passages on increasing concentrations of the fungicides (blue bars, fungicide-adapted, F). Numbers at the bottom panel show the last three digits of the natural strain’s accession number. Virulence of the strains was expressed as reduced root dry weight (RRDW, %) of infected plants compared to control plants. Different letters above the bars indicate significant differences in virulence between control (C), passed (P), and fungicide-adapted (F) strains within a single parental strain (three-sample comparison) (Mann-Whitney test with Bonferroni correction for multiple comparisons, FDR < 0.05).

Further, we investigated whether in vitro adaptation affected the virulence of the *M. nivale* strains. For this purpose, we compared the virulence of natural fungicide-sensitive strains (stored as museum cultures) with that of their in vitro-adapted fungicide-resistant derivatives, as well as with that of natural fungicide-sensitive strains that, similar to the fungicide-resistant derivatives, were consistently cultured for 20 passages in vitro but without fungicides (passaged strains). Consistent in vitro culturing without fungicide significantly reduced virulence in three (245, 615, and 624) of the five strains within the sample of strains adapted to tebuconazole (Figure 4). Herewith, compared to passaged strains, the virulence of only one strain (243) was significantly reduced following adaptation to tebuconazole. In the sample of four strains adapted to fludioxonil, two passaged strains (624 and 204) displayed reduced virulence compared to their corresponding museum strains. Compared to passaged strains, the virulence of only one strain (624) was significantly reduced following adaptation to fludioxonil (Figure 4). On average, the two assayed fungicides did not differ in how adaptation of *M. nivale* to them affected virulence levels (compared to the virulence of passaged strains) (File S4), although adaptation to each of these fungicides differentially influenced the virulence of different *M. nivale* strains (Figure 4).

### 3.4. Genome Assembly of the Natural Fludioxonil-Sensitive M. nivale Strain F_00246

**Figure 5:**
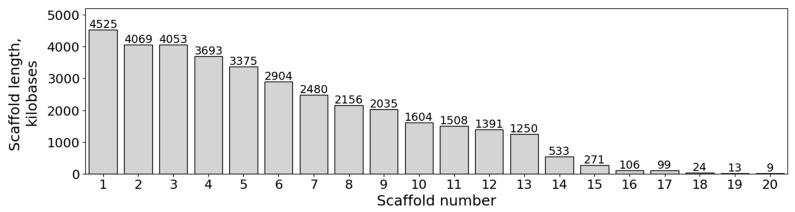
Distribution of scaffold lengths in the *Microdochium nivale* F_00246 genome assembly.

Details on the PacBio HiFi genome sequencing of the natural fludioxonil-sensitive *M. nivale* strain F_00246 are provided in Table S1. The genome size estimation of the *M. nivale* strain F_00246 using k-mer frequency analysis for k-mer lengths ranging from 21 to 31 yielded a range of approximately 33.0–33.5 Mb (Table S2). Verification of PacBio HiFi read coverage against the preliminary genome size estimate, using a mean read length of 4622 bp and 145,922 filtered reads, yielded approximately 20.13× coverage for a 33.5 Mb genome. The optimal genome assembly, selected from multiple assemblies generated under different parameterizations, spans 36.098 Mb in 20 scaffolds (N50 = 3.38 Mb; largest scaffold = 4.525 Mb, L50 = 5). Thirteen scaffolds exceeded 1 Mb in length (Figure 5). The assembly has been deposited at GenBank as a part of the NCBI BioProject PRJNA1356363. The assembly can be alternatively accessed at [59].

The genome assembly had a base-level consensus quality (QV) of 99.95%. Completeness, assessed using various BUSCO (Benchmarking Universal Single-Copy Orthologs; [67]) metrics, ranged from 98.8% to 99.5% (Table S3). Structural annotation of the *M. nivale* F_00246 genome using the Funannotate pipeline yielded 10,745 gene models, including 10,594 protein-coding genes and 151 tRNA genes.

### 3.5. Identification of Mutations That Arose During Fludioxonil Adaptation in M. nivale

Details on the Illumina genome sequencing of the fludioxonil-adapted *M. nivale* strain F_00246-FR (a derivative obtained in vitro from the natural fludioxonil-sensitive *M. nivale* strain F_00246) are provided in Table S4. Out of 16,092,182 Illumina reads generated for the *M. nivale* strain F_00246-FR, 15,645,462 (97.22%) mapped to the assembled genome of the parental *M. nivale* strain F_00246 (Section 3.4), demonstrating high mapping efficiency. A summary of alignment quality metrics following filtering and duplicate removal is provided in Table S5.

Comparison of Illumina reads from the fludioxonil-adapted *M. nivale* strain F_00246-FR against the assembled genome of the parental fludioxonil-sensitive *M. nivale* strain F_00246 identified 2039 small variants using GATK4 (1206 single-nucleotide polymorphisms (SNPs) and 833 insertions/deletions (indels)) and 1958 variants using Strelka2 (1107 SNPs and 851 indels). The consensus variants, detected by both tools, totaled 1538 (818 SNPs and 720 indels) and were considered true variants, all listed in Table S6. The distribution of the consensus set of SNPs and indels across different functional gene supercategories (derived from the reference genome assembly of *M. nivale* strain F_00608; [58]) is presented in Table 1.

In total, 127 mutations were identified in the coding regions (CDS), 124 in introns, and 1287 in intergenic regions. Mutations in CDS were classified as missense (n = 58), synonymous (n = 59), frameshift (n = 6), stop-gained variant (n = 3) and stop-lost variant (n = 1). All mutations affecting protein structure (missense, frameshift, and nonsense (stop related); 68 in total) were found within 13 genes (Table 2). Notably, the gene encoding the dynein assembly factor with WDR repeat domains 1 harbored 53 protein-affecting mutations, including one nonsense mutation at codon 295. For all other genes, no more than two protein-affecting mutations were detected within coding regions. The extended version of the table containing the interpretation of mutations is available in File S5.

## 4. Discussion

In the present study, we assessed the intrapopulation variability of *M. nivale* strains in their resistance/sensitivity to tebuconazole and fludioxonil—the fungicides recommended for controlling *M. nivale*-caused snow mold disease. No strains with a high level of resistance (to concentrations used in agricultural practice) were found among the 136 analyzed *M. nivale* strains from two populations. However, different strains within each analyzed population exhibited different levels of sensitivity to these fungicides. The less sensitive strains evidently have a higher probability of surviving after fungicide application, especially since fungicide concentrations within the agrocenosis are uneven. Although a high level of resistance to the two studied fungicides is not frequently observed in phytopathogenic fungi, some strains within several phytopathogenic species have been reported to possess a high level of resistance to tebuconazole (*Alternaria alternate* [68], *Rhizoctonia solani* [69], *Fusarium oxysporum* [70]) and fludioxonil (*Fusarium asiaticum*, *Penicillium digitatum* [71], *Fusarium graminearum* [72]). Different sensitivity levels to these fungicides have been more widely observed among phytopathogens such as *Alternaria alternate* [73], *Zymoseptoria tritici* [10,11], *Mycosphaerella graminicola* [74], *Rhizoctonia solani* [69], *Fusarium oxysporum* and *F. graminearum* [75,76], *Botrytis cinerea* [29], *Aspergillus uvarum* and *A. tubingensis* [77].

The acquisition of resistance (or reduced sensitivity) to tebuconazole or fludioxonil has been shown to differentially affect the virulence and other fungal phenotypes, even within a particular species. In *Penicillium expansum*, tebuconazole resistance negatively correlated with virulence [31], whereas in another study, strains of this species resistant to another azole fungicide (thiabendazole) were as virulent as thiabendazole-sensitive strains [30]. Tebuconazole resistance has also been shown to negatively affect the virulence of *Fusarium graminearum* [78]. Fludioxonil resistance negatively affected the virulence of *Botrytis cinerea* strains originating from strawberry and tomato [25], whereas fludioxonil-resistant strains of this species originating from grapevine were as virulent as fludioxonil-sensitive strains [28]. Similarly, in different studies, fludioxonil resistance differentially affected the virulence of *Penicillium expansum* [30,33,79]. Therefore, we assessed possible associations between the level of sensitivity of the studied *M. nivale* strains to either tebuconazole or fludioxonil and their virulence toward three winter cereal crops (rye, wheat, and triticale), as well as with their origin (the host crop, plant part, or agrocenosis from which the strains were isolated). No such associations have been revealed in our study.

The studied *M. nivale* strains have been previously divided into different phylogenetic groups based on the sequences of ITS2, EF-1α, and β-Tub [48]. In the present study, we examined whether strains from different phylogenetic groups differed in their average sensitivity level to tebuconazole or fludioxonil. Strains of one of the phylogenetic groups differentiated by the EF-1α sequence (“B” variant) were, on average, less sensitive to tebuconazole than the strains of the other one (with the “A” variant of EF-1α), whereas strains of one of the phylogenetic groups differentiated by the β-Tub sequence (“C” variant) exhibited lower sensitivity to fludioxonil than the strains of the other groups (with the “A” and “B” variants of β-Tub). Thus, our findings indicate that different phylogenetic groups of *M. nivale* have different predispositions to decreased sensitivity to tebuconazole and fludioxonil. In previous studies on other phytopathogenic species, the association between genetic features and tebuconazole/fludioxonil resistance has been analyzed only in the context of high levels of fungicide resistance conferred by mutations in the gene encoding fungicide-target proteins [10,11,20,29,69,73].

Since no *M. nivale* strains with a high level of resistance to the analyzed fungicides were found in our study, we analyzed whether the strains of this species have the potential to acquire a high level of resistance to these fungicides (similar to *Fusarium graminearum* [78], *Rhizoctonia solani* [69], *F. asiaticum* [27], and *B. cinerea* [29] species among which either fludioxonil- and/or tebuconazole-resistant strains have been described) and, if so, to compare the efficiency of *M. nivale* adaptation to fludioxonil and tebuconazole. For this, we modeled the process of fungicide adaptation in vitro by culturing the most fungicide-sensitive strains at continuously increasing concentrations of fungicides. The tested strains acquired high fungicide resistance over time, e.g., they were able to grow on fungicide concentrations used in agricultural practice. Herewith, the adaptation to tebuconazole was more effective than to fludioxonil. This indicates that fludioxonil is a more reliable fungicide for controlling *M. nivale* than tebuconazole, since the acquisition of resistance to fludioxonil proceeds less effectively. This is in line with the view that fludioxonil is regarded as a “low-risk fungicide”, meaning that adaptation to it is a rare phenomenon [20].

Five of the nine in vitro fungicide-adapted strains exhibited reduced virulence compared to their parental natural fungicide-sensitive strains. However, in this case, the observed reduction in virulence might have arisen not only due to fungicide adaptation process itself, but also because of the prolonged cultivation of the strains in vitro during the adaptation, which is known to have possible negative effects on virulence [80]. To differentiate the effect of these two factors on the reduced virulence of some of the fungicide-adapted *M. nivale* strains, their virulence was compared not only with parental fungicide-sensitive strains stored as museum cultures but also with parental strains that were, similar to the fungicide-adapted derivatives, consistently cultured in vitro but without fungicides (passaged strains). The prolonged in vitro cultivation (without fungicide) appeared to have a negative effect on the virulence of half of the tested strains. In turn, only one of the five tebuconazole-adapted strains and only one of the four fludioxonil-adapted strains exhibited reduced virulence compared to the corresponding passaged strains. These results indicate that fungicide adaptation itself has little, if any, effect on the virulence of the studied strains. Therefore, the reduced virulence observed in more than half of the fungicide-adapted strains compared to the corresponding museum strains is likely related not to the acquisition of fungicide resistance itself, but because the selection factor was only fungicide, not the ability to parasitize a host plant. This is in agreement with the lack of association between the level of sensitivity to tebuconazole or fludioxonil and virulence observed in the sample of 136 natural strains of *M. nivale*.

The molecular mechanisms of adaptation to tebuconazole are relatively well studied across different species and are primarily associated with mutations in the lanosterol 14α-demethylase gene (CYP51) [7,8,10], increased expression of this gene, or overproduction of transporters that remove the fungicide from the cell [8,9,10]. The mechanisms of adaptation to fludioxonil, which have been shown to involve mutations in genes encoding various kinases involved in the osmotic stress response, are much less extensively studied and understood [16,17,18,19,20]. To get closer to understanding the mechanisms of fludioxonil adaptation in *M. nivale*, the whole genome sequence of a fludioxonil-adapted in vitro strain, F_00246-FR (a fludioxonil-resistant derivative), was compared to that of its parental natural fludioxonil-sensitive strain, F_00246, in order to identify mutations that arose during the development of resistance.

Sequencing of genomes of the F_00246 and F_00246-FR strains and their comparison revealed 1538 mutations arose following fludioxonil adaptation. Most of the mutations (around 92%) were located in non-coding regions. In CDS, 127 mutations were identified, of which 68 affect the primary structures of 13 proteins. Mutations in the genes of three of these proteins, in our opinion, merit particular attention regarding their potential role in adaptation to fludioxonil.

First, a missense mutation was observed in the *ssk2* gene, which is annotated as encoding a mitogen-activated protein kinase kinase kinase (MAP3K). The protein encoded by this gene, among well-annotated proteins from the NCBI database, is most similar to the OS-4 MAP3K of *Podospora didyma* (accession number KAK3394760.1), with 100% coverage and 62.1% identity to SSK2 from *M. nivale* F_00246, as well as to the SSK2 serine/threonine-protein kinase of *Verticillium dahliae* (accession number XP_009649399.1), with 100% coverage and 63.0% identity to SSK2 from *M. nivale* F_00246. Importantly, knockout of the *ssk2* gene in *V. dahliae* has been shown to increase resistance to fludioxonil and cause hypersensitivity to high osmolarity [81]. The SSK2 MAP3K (OS-4 MAP3K) is a component of the high-osmolarity glycerol (HOG) pathway, which is known to be targeted by fludioxonil to induce hyphal swelling and bursting. This explains why knockout of this gene leads to increased fludioxonil resistance in *V. dahliae*. Therefore, the missense mutation in the *ssk2* gene in *M. nivale* following fludioxonil adaptation might also reduce fludioxonil-mediated activation of the HOG pathway, leading to increased resistance to this fungicide.

Second, after *M. nivale* adaptation to fludioxonil, a missense mutation was identified in the gene encoding benzoate 4-monooxygenase. The primary structure of this *M. nivale* enzyme displays similarity (85% cover and 44.2% identity) to the *Fusarium verticillioides* CYP57A1 enzyme (RBQ95979.1), which is known for its ability to degrade the herbicide fomesafen, which, similar to fludioxonil, has an aromatic nature [82]. Therefore, it can be hypothesized that the mutation identified in the benzoate 4-monooxygenase gene of *M. nivale* during fludioxonil adaptation may enhance the enzyme’s ability to degrade fludioxonil. However, this hypothesis requires targeted functional validation.

Third, an exceptionally high mutation rate (53 protein-affecting mutations) was found in a gene annotated as encoding a dynein assembly factor with WDR repeat domains 1, which, based on its domain organization, is a heterokaryon incompatibility (HET) protein. HET proteins prevent the development of heterokaryotic mycelium when hyphae from vegetatively incompatible strains fuse [83]. Multiple mutations in genes encoding HET proteins, particularly the so-called Repeat-Induced Point (RIP) mutations, which predominantly occur in interdomain regions (similar to those observed in our study on *M. nivale*), have been previously described in fungi, including *Podospora anserine* and *Neurospora crassa* [84,85]. A high mutation rate in *het* genes is thought to facilitate a variety of crosses among different genotypes, thereby enhancing the adaptability of the species under adverse conditions [86,87]. Therefore, modifications of the HET protein driven by multiple mutations in *M. nivale* during fludioxonil adaptation are unlikely to have a direct effect on fludioxonil resistance; however, they may facilitate increased gene flow during sexual reproduction, promoting the emergence of resistant variants. Additionally, despite the large number of *het* genes in the *M. nivale* genome (133 *het* genes; [58]), it is quite surprising that mutations (specifically multiple mutations) during fludioxonil adaptation were observed in only one of these genes. Why mutations arose specifically in this particular *het* gene and not in any of the other 132 *het* genes during *M. nivale*’s fludioxonil adaptation remains to be determined.

## 5. Conclusions

No strains with high resistance levels to the fungicides fludioxonil and tebuconazole were found within the studied Russian populations of the snow mold causal fungus, *Microdochium nivale*, although some intrapopulation diversity was observed in sensitivity to low doses of these fungicides. The sensitivity levels to fludioxonil and tebuconazole were not associated with the virulence of the studied strains. *M. nivale* strains from different phylogenetic groups, differentiated by sequences of the elongation factor 1α and β-tubulin genes, exhibited different predispositions to decreased sensitivity to tebuconazole and fludioxonil. *M. nivale* strains can adapt to high concentrations of fludioxonil and tebuconazole in vitro with little or no effect on virulence, indicating a potential risk of rapid resistance development under field conditions. In vitro adaptation to fludioxonil was significantly less effective than to tebuconazole, suggesting that fludioxonil is a more reliable fungicide for controlling *M. nivale*-induced snow mold than tebuconazole. Fludioxonil adaptation in *M. nivale* is associated with multiple mutations in the genome. Mutations in the coding sequences of genes encoding mitogen-activated protein kinase kinase kinase, benzoate 4-monooxygenase, and HET protein are proposed as top candidates contributing to fludioxonil resistance in *M. nivale*.

## Figures and Tables

**Table 1:** Distribution of single nucleotide polymorphisms (SNPs), insertions and deletions (indels) that arose following *Microdochium nivale* adaptation to fludioxonil across different functional gene supercategories (derived from the reference genome assembly of *M. nivale* strain F_00608; [58]).

Supercategory	SNP	Deletion	Insertion
Cytoskeleton	141	1	1
Other	15	19	0
Plant cell wall modification	0	3	0
Primary metabolism	2	15	0
Secondary metabolism	11	6	0
Signaling	1	1	0
Stress-related	0	5	0
Transcription factors	0	2	0
Transport	0	9	1
Unknown	2	10	0
intergenic	646	622	19
Non-annotated genes	0	6	0

**Table 2:** *Microdochium nivale* F_00246 genes harboring mutations mediated by fludioxonil adaptation that affect protein structure (SNP—single nucleotide polymorphism, In—insertion, Del—deletion). Gene ID prefixes were assigned according to the Funannotate pipeline.

Gene	Annotation	SNP	In	Del
FUN_004967	dynein assembly factor with WDR repeat domains 1	53	0	0
FUN_008595	benzoate 4-monooxygenase	2	0	0
FUN_010062	hybrid polyketide synthase	2	0	0
FUN_005818	FxSxx-COOH system tetratricopeptide repeat protein	2	0	0
FUN_000391	solute carrier family 27 (fatty acid transporter) member 1/4	0	1	0
FUN_003866	hypothetical protein	1	0	0
FUN_002296	hypothetical protein	0	0	1
FUN_001727	ankyrin repeat domain-containing protein 50	0	1	0
FUN_005150	lysophospholipase-like protein	0	0	1
FUN_007452	FxSxx-COOH system tetratricopeptide repeat protein	1	0	0
FUN_008898	methyltransferase domain-containing protein	0	0	1
FUN_000651(MRPL9)	large subunit ribosomal protein L3	0	0	1
FUN_003478 (SSK2)	mitogen-activated protein kinase kinase kinase	1	0	0

## Data Availability

Sequencing data from this study can be found in the National Center for Biotechnology Information (NCBI) Sequence Reading Archive (SRA) under the PRJNA1356363 bioproject.

## Supplementary Materials

The following supporting information can be downloaded at: https://www.mdpi.com/article/10.3390/jof11120859/s1, File S1: Spearman correlation coefficients between the sensitivity levels of *Microdochium nivale* strains to fludioxonil and tebuconazole; File S2: Sensitivity levels of *Microdochium nivale* strains, originating from different agrocenoses (Arsk and Laishevo), to fludioxonil and tebuconazole; File S3: Sensitivity levels of *Microdochium nivale* strains, originating from different crop hosts (winter rye, winter wheat, and winter triticale) or different plant parts (healthy parts of shoots (HPS), roots, or dead parts of shoots (DPS)), to fludioxonil and tebuconazole; File S4: Relative reduction in virulence of *Microdochium nivale* strains following in vitro adaptation to fludioxonil or tebuconazole; File S5: The distribution of single nucleotide polymorphisms (SNPs), insertions and deletions (indels) across different functional gene supercategories (derived from the reference genome assembly of *Microdochium nivale* strain F_00608; Table S1: Details on the PacBio HiFi genome sequencing of the fludioxonil-sensitive *Microdochium nivale* strain F_00246; Table S2: Genome size estimation of *Microdochium nivale* strain F_00246 using k-mer frequency analysis; Table S3: Assessment of the *Microdochium nivale* F_00246 genome assemly completeness using BUSCO: comparison across AUGUSTUS, MetaEuk, and miniprot; Table S4: Details on the Illumina genome sequencing of the fludioxonil-adapted *Microdochium nivale* strain F_00246-FR (in vitro derivative of the fludioxonil-sensitive strain F_00246); Table S5: BAM quality metrics for the alignment of Illumina reads obtained for fludioxonil-resistant *Microdochium nivale* derivative (F_00246-FR) against the genome assembly of the natural parental fludioxonil-sensitive *M. nivale* strain F_00246; Table S6: Mutations revealed in *Microdochium nivale* following fludioxonil adaptation.
